# Comprehensive mental health and psychosocial support for war survivors at Chenna Kebele, Dabat woreda, North Gondar, Ethiopia

**DOI:** 10.1186/s12888-023-04653-8

**Published:** 2023-03-16

**Authors:** Niguse Yigzaw, Tewodros Hailu, Mekides Melesse, Ashenafi Desalegn, Haymanot Ezezew, Tebaber Chanie, Goshu Nenko, Moges Tesfahun, Simegn Sendek, Seblewongel Tinsae

**Affiliations:** 1 Department of Psychiatry, College of Medicine and Health Science, University of Gondar, P. O. Box – 196, Gondar, Ethiopia; 2 Department of Psychology, College of Social Science and Humanity, University of Gondar, Gondar, Ethiopia; 3 Department of Sociology, College of Social Science and Humanity, University of Gondar, Gondar, Ethiopia; 4 Department of Social Anthropology, College of Social Science & Humanity, University of Gondar, Gondar, Ethiopia

**Keywords:** War survivor, Mental health, Psychosocial support, Social networking

## Abstract

**Background:**

Armed conflict and natural disasters cause serious psychosocial problems. Providing comprehensive bio-psychosocial support to the community after a war, traumatic, or devastating event has a significant impact on community reconstruction and resilience. As a result, the goal of this project was to conduct community diagnosis, identify individuals experiencing psychological distress, and provide comprehensive mental health and psychosocial support for the Chenna Kebele population in Dabat Woreda, North Gondar, Ethiopia.

**Methods:**

A mixed research approach, specifically an explanatory study design, was used to assess psychosocial issues among war survivors. In-depth interviews, focus group discussions, and observation were used to assess the psychosocial and economic consequences of the war on survivors. Additionally, a structured questionnaire was employed to assess mental health problems among the target population. The project was conducted in three phases.

**Result:**

A total of 550 households were assessed using a structured questionnaire. Of them, 45 people reported a range of mental health issues, including post-traumatic stress disorder (PTSD), major depressive disorder (MDD), adjustment disorder, protracted bereavement disorder, and insomnia. PTSD is the most common diagnosis, accounting for 38 cases. Four cases of major depressive disorder, three cases of prolonged grief, and two neurologic cases were also identified during the screening. Females had a higher number of PTSD cases as compared with males. Fifteen women reported sexual violence, and the number may be high because of underreporting and refusal to disclose the situation. The result also indicated significant property damage, loss of life stocks, and disengagement from basic services like water. Intervention with follow-up was provided at the individual, group, and community levels in order to reverse the devastating situation. The intervention included pharmacotherapy, psychotherapy, and social networking.

**Conclusion and recommendation:**

Overall, the community has experienced multiple psychosocial and economic problems. Hence, providing holistic mental health psychosocial support, clearing the site and burying the dead body, and re-initiating the terminated social gathering event will alleviate the existing problem and create a resilient community.

**Supplementary Information:**

The online version contains supplementary material available at 10.1186/s12888-023-04653-8.

## Introduction 

Following the peaceful transition of power among Ethiopian government officials, there were some disagreements among political leaders as well as regional and federal government officials. Long-term armed conflicts continue to result from such disagreements. Overall, the incidents of armed conflict in Ethiopia are markedly increasing. In particular, the yearlong war in the northern region of Ethiopia has caused a disaster in the country. Many have lost their lives, been injured, been sexually violated, and have lost their properties. Due to these, survivors of the war have experienced a serious psychological and social crisis. When the war expanded to the Amara region, the battles took place in different areas, and Chenna Kebele was among the deadly battling grounds.

The TPLF took the village of Chenna Teklehaymanotin in the Amhara region's Dabat district between August 31 and September 4.

According to Amnesty International's report [1], the armed group killed hundreds of residents; gang-raped dozens of women and girls, and looted and destroyed private and public property during the occupation. Authorities put the civilian death toll at 200, with many more still missing. Similarly, doctors at the nearby hospital estimated that at least 125 people had died [2]. The Ethiopian Human Rights Commission also discovered 47 individuals who had been murdered extra-judicially by the armed group, with some of the bodies discovered with their hands tied behind their backs [3]. During the takeover, TPLF soldiers raped and sexually molested about 30 women and girls between the ages of 14 in and around Chenna [1].

There is enough data to suggest that communities that have endured major military conflict have a high prevalence of mental suffering [4–6]. According to research, war victims have a higher rate of PTSD (post-traumatic stress disorder), sadness, anxiety, and somatization [7, 8, 6]. The chronic challenges in their everyday lives that are caused or aggravated by armed conflict will influence the intensity of the victims' psychological suffering. Death or separation of family members; bodily injuries; sexual violence; property destruction; safety and security concerns; destruction of social networks; and anxiety about the future are just a few examples [9]. To mitigate the long-term effects of such armed violence on the community's healthy lifestyle, extensive mental health, and psychosocial care are required.

A professional team from the University of Gondar was sent to collect preliminary data on the overall psychosocial and economic impact of the armed conflict in and around Chenna Kebele. The report showed increased traumatization among people who survived the tragic experience, witnessed the deaths of family members, and were victims of sexual violence. The team also observed different places that were covered by dead bodies, and some of the bodies were buried within the houses of the village residents. The Associated Press [10] also reported unburied corpses at the scene, and some of the bodies were in military clothing.

The war targeted the homes of civilians too, in which many houses were destroyed and a lot of livestock animals were killed. People are experiencing severe grief, guilt for not being able to defend family members, loneliness (particularly for those who have experienced sexual violence), anger, uncertainty, or worry about future threats as a result of the aforementioned problems and losses. People in the community are also experiencing a terrible psychological and social crisis as a result of unmet economic and fundamental requirements.

In addition, those who fled to the neighboring places say that coming back to their home and facing their loss is considered hard and impossible, as expressed by most of the people who are displaced. Even though the majority of people are currently returning to their homes, some are hesitant to do so due to flashbacks from their direct exposure to traumatic events. Moreover, social support systems like 'Mahber,’ ‘Edir,’ and ‘Equb’ (the names are in the local language), were significant protective factors in enhancing the resilience of community members to cope with crises. Unfortunately, many of these social networks are dysfunctional, so survivors weren’t able to share their pain, grievances, and losses.

After discussing the issue with authorities from the University of Gondar and Dabat Wereda, an agreement was reached to respond quickly and offer victims psychosocial care. A professional team comprised of psychiatry, psychology, social work, sociology, and social anthropology was formed and immediately engaged in designing and implementing a multi-layered comprehensive intervention based on Inter-Agency Standing Committee (IASC) guidelines for mental health and psychosocial support in an emergency setting [11].

Hence, the current study applies a community-based intervention with an asset-based community development model, to provide comprehensive mental health and psychosocial support. Focusing on intervening severe mental health problems, providing psychosocial support for sexual violence survivors, and re-establishing the social network to enhance resilience.

## Methods

### Research design

In this study, a mixed research design was used to assess post-war psychosocial problems among survivors and evaluate the outcome of comprehensive mental health and psychosocial support for the victims.

### Study area and period

The project was conducted at Dabat woreda, Chenna Keble, from December 1, 2021, to January 30, 2022. Dabat is a town in northern Ethiopia. Located on the Semien Mountains along the Gondar-Debark highway it is in the Semien Gondar Zone of the Amhara Region. Dabat is one of two towns in Dabat woreda. Chenna is one of the Kebele in Dabat Woreda, where TPLF occupied for five days from August 31 to September 31. During the occupation, the armed group killed hundreds of residents, raped dozens of women and girls, and looted and destroyed private and public properties.

### Population and sampling procedures

The study population consisted of adults over the age of 18, with a focus on those who reported mental disorder symptoms. Accordingly, purposive sampling techniques were used to select participants with signs and symptoms of mental distress, and at the household level for focus group discussion.

### Data collection procedure

Data were collected using a semi-structured questionnaire that used DSM-5 criteria to assess common mental disorders such as PTSD, Anxiety, Depression, and Psychosis. A group of professionals from the departments of psychiatry, clinical psychology, and social work was involved in the data collection process.

The project was divided into three phases: screening and identification, intervention, and follow-up and referral linkage. At the individual level, data were gathered through intake interviews, observations at the health post, and home-to-home visits conducted throughout the kebele. Additionally, to identify common problems related to social networking and integration, a focused group discussion was held with religious and community leaders, as well as kebele and woreda administrators. Furthermore, the action plan was discussed with the leaders to increase community cooperation and active engagement, and to assure the cultural appropriateness of the activities. The post-test data were gathered through follow-up interviews and observations at the individual and community levels.

### Intervention plan

**Fig. 1:**
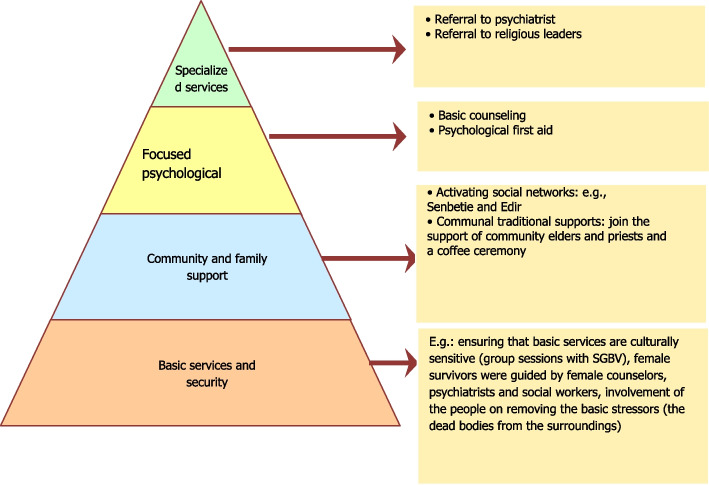
Intervention pyramid for mental health and psychosocial support in emergencies as IASC guidelines

The intervention will be provided using the "mental health and psychosocial support in an emergency setting guideline" that was developed by the Inter-Agency Standing Committee (2007) as presented in Fig. 1.

Detailed intervention strategies were developed after the preliminary assessment. Based on the data gained from the assessment, individual and community-level mental health and psychosocial support was provided as shown in Table 1.

**Table 1:** List of actions and activities performed by professionals from psychiatry, psychology, social worker and sociology during the implementation of the service at Chena kebele, Dabat woreda December 1, 2021 to January 30, 2022

	**Major tasks**	**Sub Activities**
1	Coordination	- Contacting focal persons and key informants in the area - Establish discussion with stakeholders; including religious and community leaders, and local authorities.
2	Screening and problem identification	- Conducting initial assessment and observation at individual level - Home to home visit - Focused group discussion with religious and community leaders, and local authorities
3	Mental health services	- Provision of psychiatric medication for people with acute stress - Individual and group counseling for people with mild reactions - Training for paraprofessionals on screening, provision of psychological first aid, and referral mechanism for severe cases.
4	Community mobilization	- Community education on the impact of war on people’s mental health and psychosocial well-being - Discussion with religious fathers on their role in assisting the healing process - Reinstalling social support systems (‘Mahber’, ‘Ekub’ and ‘Edir’) by discussing with community leaders. - Enhancing social integration and gathering through cultural activities like coffee ceremony
5	Consultancy	- Discussion with zonal and woreda health office on the medical support that the community demand - Discussion with zonal and woreda education office on restoring the destroyed materials, and assist students and teachers to continue with teaching and learning process at primary school - Discussion with the office of women, youth and children affair on helping women and girls that experienced GBV, and children and youth that are subject to the traumatic experience
6	Dissemination of information	- Providing information for the community about the available medical services and health insurance by collaborating with governmental or non-governmental organizations that works on health sectors - Giving information on how to access basic need support by linking them the concerned body.

### Ethical consideration

An ethical approval letter was obtained from the University of Gondar Institutional review board with reference Number VP/RTT/05/969/2022. All methods were carried out in accordance with relevant guidelines and regulations including ICH-GCP and the declaration of Helsinki. A formal letter of support was submitted to each woreda office and stakeholders. Written informed consent was taken from each participant after an explanation of the objective of the study. Participants had the right to withdraw at any time during the study period. Privacy and confidentiality of the response were maintained. Cases with psychological distress were linked to professionals for further investigation and therapy.

## Result 

A total of 550 households were assessed, and 46 of them reported certain types of mental health problems. Thirty-one cases (68.9%) were reported by females, while the remaining fourteen (31.1%) were reported by males. The average age is 43, with the most commonly diagnosed ages ranging from 25 to 39. In terms of diagnosis, PTSD was found to be the most prevalent among the cases, accounting for 84.4%, followed by major depressive disorder which accounted for 8.9. The additional case was insomnia, psychosis, and prolonged grievance.

Regarding gender and age distribution, 24 (63.2%) cases of PTSD were reported by females, while 7 (36.8%) were reported by males. Approximately 35 (92.1%) of the PTSD cases reported were by individuals aged 25–39, with the remaining 3 (7.9%) reported by the younger population. Depression was also found to be common among females, with 3 (75%) of cases reported by women and the remaining 1(25%) reported by men. In contrast to PTSD, depression cases were mostly reported by elders that ranged from 55 to 69 of age. However, while prolonged grievance was reported by women, insomnia and psychosis cases were reported by men.

According to the interview data, the individuals who reported symptoms of mental disorders were in the area of the war during active combat, some of them witnessed a family member being executed in front of them, some experienced a death threat, and some reported losing four to six family members. As indicated in Table 2.

**Table 2:** Socio-demographic and diagnosis distribution of war survivor Chenna kebele, Dabat woreda, 2021

*Variable*	*Categories*	*Frequency*	*Percentile (%)*
Gender	Male	14	31.1
	Female	31	68.9
Age	18–24	3	6.7
	25–39	20	44.4
	40–54	10	22.2
	55–69	10	22.2
	70 <	2	4.4
Diagnosis	PTSD	38	84.4
	Major Depression Disorder	4	8.9
	Others	3	6.7

Regarding sexual violence, during the armed conflict, many women and girls have suffered sexual violence, including gang rape. Fifteen women reported sexual violence. The number of incidents is expected to be exceeded, as one of the victims said: “similar things happened to a lot of women who were trapped in the area during the war, but only a few of us came forward and sought help, despite the serious social stigma and discrimination.” Consequently, most of the victims complained of sleeping problems, flashbacks, and a fear that the incident could happen again. And three of the victims were diagnosed with a sexually transmitted infection, but all tested negative on the HIV test.

Apart from mental health problems, individuals reported the following physical complaints: pain related to injuries, eye problems, severe headaches, and pain associated with abdominal pain. Others, especially those taking medications for chronic illnesses, also have no access to the medication.

Concerning socioeconomic issues, data from interviews and observations revealed that properties, including houses, had been completely destroyed by heavy weapons, with bullets piercing the roof as shown in Picture 1.

Many livestock were also killed during the war, and crops were damaged before harvesting, making the community's living conditions even more difficult. On the other hand, because many community members have fled and been displaced to other areas, the social systems that are used to support members during crises are not functioning. As a result, the number of social networks in which communities support one another has decreased.

Members of the community have also complained about the presence of corpses in fields, around houses, in water tankers, and in rivers that continued to traumatize people, causing them not to return home, drink water from those reservoirs, or even collect edible crops from their backyard. Women and children are particularly affected by unburied bodies; women who witnessed the dead bodies while fetching water, passing through neighborhoods, or in their backyards developed symptoms of depression and anxiety. Furthermore, some children are terrified of corpses, while others play freely in the yard. Both abnormal reactions can have an impact on children's psychological and personality development.

Additionally, the schools in chenna and the surrounding are used as a camp by the armed group and subjected to damage, which has hindered the recovery process of children.

### Individual-level intervention

After the screening and diagnosis, people were treated with pharmacotherapy and psychotherapy based on the severity of the symptoms. Accordingly, all of them were treated and received medications that include Amitriptyline, Diazepam, Chlorpromazine, and Fluoxetine, in collaboration with the psychiatry team of the International Medical Corps (IMC). Counseling and psychotherapeutic services were provided for individuals with mild to moderate symptoms, and for those with a protracted grievance. The counseling approach was crisis-focused and based on the nature of individual complaints, and it was very brief, lasting for two weeks including the follow-up. Techniques like normalization, affirmation, and empathy are used to enhance interaction and understand their situation. The counseling also included interpersonal therapy that focused on helping the survivors share their experiences with others and engage in collective cultural and spiritual activities that will assist in their relief (coffee ceremonies, visiting religious fathers, and praying together).

Group therapy was given to the SGBV survivors. The victims’ ages range from 15 to 25, and most of the victims seem resilient since they have received psychosocial support from different stakeholders. Considering this, the group therapy focused on experience sharing, which helped the victims learn that their reactions are normal to such violence, and the sessions have assisted victims in sharing possible coping mechanisms with each other. The role of the counselor was to facilitate the discussions and empathize with the client's experiences. However, disclosing the incident was very challenging for the victims. During the group session, a girl explained that she had been bitten by her parents to lie and tell people that she was not raped, and her parents also threatened the social worker. The survivors are facing social exclusion, so they moved to a town near Chena. Some of them have started their studies; others are trying to get some work and lead their lives. The girls have established a support group that they will organize and that will meet weekly to discuss different issues.

Finally, to sustain the mental health and psychosocial support provision in the setting, training was provided to participants from the different sectors at Chena Kebele. The training was given to 21 people that have everyday contact with the community, which include health extension workers, teachers, agriculturalists, priests, community elders, and social workers. The training focused on creating awareness about post-crisis mental health problems and the provision of psychological first aid. Promoting social networks, community mobilization, and community education were among the training focuses. After the training, the paraprofessionals were introduced to the community at church during the community education for the people to be able to know them as shown in Picture2*.*

### Community-level intervention

These interventions are intended to raise community awareness of the psychological, social, and economic consequences of wartime conflict. Hence, various activities were performed, including community psycho-education, which aimed to increase understanding of mental health problems and available support. The psycho-education was provided through door-to-door visits and mass education that take place at church during Sunday gatherings. The education focused on psychological help and social support for the needy, bringing back peace, stability, and trust within the community, and the role of the community members to help the survivors without stigma and discrimination, as shown in Picture 3*.*

A discussion with priests was the other activity performed, which focused on the role of the religious father in assisting the recovery process of the community. The team recommended activities such as blessing the cursed areas so that the community members could fetch water and collect crops to feed themselves. In addition, counseling people who are still grieving through religious confession and changing unhealthy thoughts through guidance. Furthermore, the team discussed the issue of SGBV survivors, the social isolation they are experiencing, and the mechanisms to reintegrate them into the community. Finally, restoring dysfunctional social networks (‘Senbete’, ‘Edir’, ‘Ekob’, ‘Debo’, and ‘Mahiber’) was the other discussion point to support the social healing process, as shown in Picture 4*.*

The team organized a coffee ceremony, which is a traditional practice where people gather around to discuss social issues while drinking coffee. Thus, a coffee ceremony was prepared around the house of their former leader, who passed away during the war. The team invited neighbors and bypasses. The community members discussed bringing back their social life or social gatherings like ‘Senbetie’ and ‘Mahber’. The community members spent some time at the coffee ceremony discussing each other.

### Resource mobilization

The team has consulted with different governmental and non-governmental offices to provide a holistic response to the community’s needs. Accordingly, there was a meeting and discussion with kebele and woreda authorities on means to replace looted and destroyed properties, especially roof covers that were pierced by bullets during the war, since they faced many problems during the rainy season and were the source of recalling the traumatic event. As a result, the team informed the relevant body of the urgency and discussed the mechanism for identifying potential stakeholders who could donate such materials. Similarly, the team has discussed and suggested a solution for a proper burial of dead bodies immediately, which is traumatizing the residents, particularly women and children.

The team has also consulted with the health bureau and NGOs for harmonized and collaborative health services. The team met with the educational bureau to get data on the damage caused and discuss immediate solutions for schools reopening, and the data showed that most of the schools are partially damaged; blackboards and desks are materials that are mainly damaged, and supportive units like libraries and laboratories are also damaged, So to immediately restore these materials, the team agreed to communicate with the university, colleges, and schools that are not affected by the war to support the schools, and as result, many books and blackboards were collected and distributed to the schools, as shown in Picture 5*.*

Among the major areas of support in an emergency setting are the provision of information and the linkage of the needy with the support provider. So, the team provided key information on how to find support and created a link between those who needed help and the service provider. For instance, during the home-to-home visit, most of the people had physical complaints, but they did not go to the health post because they did not have money to pay when the health post provides free services. After discussing with service providers, the team provided information on the available services at the health post. Additionally, most of the residents and elders, in particular, complain about eye problems, so the team communicated with the department of optometry, and they sent a team that helped more than 70 individuals. Moreover, the team linked individuals who finished psychiatric medication with NGOs working in the area, like International Medical Corps (IMC) teams, for further follow-up and evaluation.

### Follow-up and outcome

Individual and community-level follow-up was carried out through interviews and observation. Individual cases were monitored to assess progress and control side effects, and social system functionality was evaluated to assess community integration and interaction.

As a result, participants reported a considerable reduction in symptoms, and the observation revealed that patients were making good progress, with more positive results observed among those who received both psychotherapy and medicine. Individuals who received the treatment reported alleviation from emotional distress and worries, and improved social interact and sleep routine. In terms of community integration, the majority of the displaced members returned to their homes, and there was a rise in cultural and religious events involving a large number of the members. Furthermore, the majority of social ties and groupings are functionalized. The school, on the other hand, has reopened, and interested individuals and organizations are donating resources to help rebuild the school.

## Discussion

As the aim of this project is to identify psychosocial problems among war survivors and provide comprehensive mental health and psychosocial support, 45 community members reported different types of mental health problems. PTSD was found to be the most prevalent case among the survivors, along with major depressive disorder and adjustment disorder. Similar findings were reported by research conducted in Sub-Saharan African countries, Afghanistan, and Syria [12–14]. The finding of this study revealed that individuals who have been in the area throughout the war conflict, witnessed traumatic events, were threatened, and lost family members are highly prone to develop severe mental health problems, which also supported by previous researches [12, 14]. Regarding gender differences, females reported a higher number of PTSD and adjustment disorder cases as compared to males, this finding is supported by a meta-analysis conducted by Morina N, Stam K, Pollet TV, and Priebe S. [15]. Moreover, it can be explained by the tendency of a female to be victimized sexually, emotionally, and physically during crises, and the higher trend of expressing fear and emotion among females, which enhance their chance of reporting problem and seeking help. On another hand, older age has been associated with reporting depression among the cases, this mainly can be due to deep grievance, social isolation, and self-blaming as age increases.

Sexual violence against women in war and its aftermath can have almost inestimable short and long-term negative health consequences. These included risk for sexually transmitted infections (STIs) including HIV/AIDS. Furthermore, victims of sexual violence are experiencing high levels of psychological distress and mental disorders [7]. Similarly, the victims of sexual violence have reported common mental distress symptoms such as flashbacks, nightmares, guilty feelings, and social isolation, and some of them have been diagnosed with sexually transmitted infections.

Therefore, providing mental health and psychosocial support will have an eminence role in supporting the recovery process of those who suffered from mental health problems, and reinstating the social system in order to build a resilient community [11]. Accordingly, the comprehensive intervention provided at all levels has brought changes in alleviating emotional distress, and rebuilding institutions and social networks. 

## Conclusion

In general, war survivors in Chenna kebele have serious mental health problems and are in a socioeconomic crisis. The impact of armed conflict on females and children is twofold. Therefore, identifying the root causes of the community and individual problems, and providing multilevel intervention, will thereby reduce the burden of the issues. Multi-sector intervention and stakeholder collaboration to reverse the current situation necessitate resource mobilization, information exchange, and close follow-up. The restoration of social networking and gathering systems is critical to the reintegration of community everyday life. Apart from financial and material support, mental health and psychosocial support following a traumatic event have a significant positive impact on survivors.

## Recommendation

- ➢ Woreda and zonal administrative office shall focus on the stressors, social and family networking system among survivors since it has a key role in reintegrating the community and creating a resilient society.
- ➢ Governmental and non-governmental organizations need to consider comprehensive health care management focusing on mental health and psychosocial support and applying the bio-psychosocial model during an emergency.
- ➢ Local authorities should design a system to mobilize resource and enhance collaboration within and between stakeholders to rebuild institutions and reinstate basic services
- ➢A further cohort study is needed to see the long-term impact of the war on survivors, especially on children.

## Supplementary Information

**Additional file 1: Picture 1.** Sample destructed roof during the war at chenna Kebele,dabat woreda, North Gondar,Ethiopia. **Picture 2.** Training for community leaders, clergymen, woreda and kebele administrators and health extension workers on mental health and psychological support at Chenna kebele, Dabat woreda, Ethiopia 2021. **Picture 3.** Mass education on social networking and grief management at Chenna Abune Tekle Haymanot Church 2021. **Picture 4.** Restoration of Social networking following civil war at Chenna Kebele,North Gondar, 2021. **Picture 5.** Destructed class room and black board during the war at chenna Kebele primary school, Dabat woreda, Ethiopia 2021.

## Data Availability

Data will be available upon request and the IRB of the University of Gondar approves data sharing.

## Abbreviations

DSM: Diagnostic and statistical manual of mental Disorders
FGD: Focused group discussion
GBV: Gender-based violence
IASC: Inter-Agency Standing Committee
IMC: International medical corpus
MHPSS: Mental health and psychosocial support
NGO: Nongovernmental organization
PTSD: Post-traumatic stress disorder
TPLF: Tigrayan people liberation front
UoG: University of Gondar
