# Supplementary material for: Comprehensive mental health and psychosocial support for war survivors at Chenna Kebele, Dabat woreda, North Gondar, Ethiopia
Source: BMC Psychiatry. 2023 Mar 16;23:172. doi: 10.1186/s12888-023-04653-8 (PMC10018845; doi:10.1186/s12888-023-04653-8)
Supplement: Supplementary file 1 — Additional file 1: Picture 1. Sample destructed roof during the war at chenna Kebele,dabat woreda, North Gondar,Ethiopia. Picture 2. Training for community leaders, clergymen, woreda and kebele administrators and health extension workers on mental health and psychological support at Chenna kebele, Dabat woreda, Ethiopia 2021. Picture 3. Mass education on social networking and grief management at Chenna Abune Tekle Haymanot Church 2021. Picture 4. Restoration of Social networking following civil war at Chenna Kebele,North Gondar, 2021. Picture 5. Destructed class room and black board during the war at chenna Kebele primary school, Dabat woreda, Ethiopia 2021. [file 12888_2023_4653_MOESM1_ESM.zip › Picture/Picture 5.docx]

**
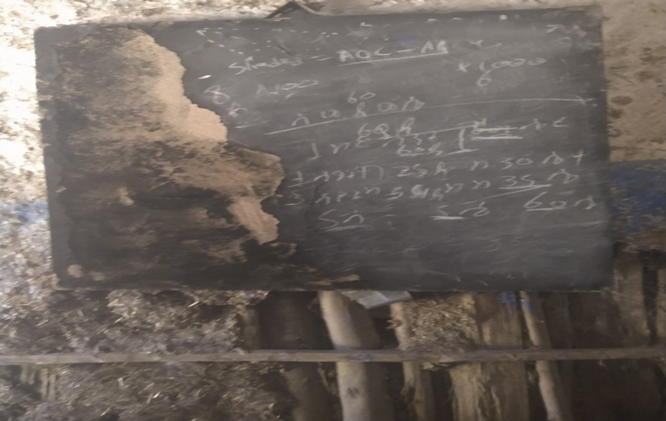
**

*Picture 5. Destructed class room and black board during the war at chenna Kebele primary school, Dabat woreda, Ethiopia 2021.*
